# Erratum: Ju, Z., et al. Integrative Analyses of Multilevel Omics Reveal Preneoplastic Breast to Possess a Molecular Landscape That Is Globally Shared with Invasive Basal-Like Breast Cancer (Running Title: Molecular Landscape of Basal-Like Breast Cancer Progression). *Cancers* 2020, *12*, 722

**DOI:** 10.3390/cancers12103062

**Published:** 2020-10-20

**Authors:** Zhenlin Ju, Anjana Bhardwaj, Matthew D. Embury, Harpreet Singh, Preethi H. Gunaratne, Isabelle Bedrosian, Jing Wang

**Affiliations:** 1 Department of Bioinformatics and Computational Biology, Houston, TX 77030, USA; zju@mdanderson.org; 2 Department of Breast Surgical Oncology, The University of Texas MD Anderson Cancer Center, Houston, TX 77030, USA; abhardwaj@mdanderson.org (A.B.); MDEmbury@mdanderson.org (M.D.E.); s.harpreet20@gmail.com (H.S.); 3 Department of Biology and Biochemistry, University of Houston, Houston, TX 77030, USA; phgunara@Central.UH.EDU

There is an error in the title of the paper [1]. The authors thus wish to make the following correction to this paper [1]:

Change the title from “Integrative Analyses of Multilevel Omics Reveal Preneoplastic Breast to Possess a Molecular Landscape That is Globally Shared with Invasive Basal-Like Breast Cancer (Running Title: Molecular Landscape of Basal-Like Breast Cancer Progression)” to “Integrative Analyses of Multilevel Omics Reveal Preneoplastic Breast to Possess a Molecular Landscape That Is Globally Shared with Invasive Basal-Like Breast Cancer”.

The authors would like to apologize for any inconvenience caused to the readers by this change. The original article has been updated.
