# Supplementary material for: Integrative Analyses of Multilevel Omics Reveal Preneoplastic Breast to Possess a Molecular Landscape That is Globally Shared with Invasive Basal-Like Breast Cancer (Running Title: Molecular Landscape of Basal-Like Breast Cancer Progression)
Source: Cancers (Basel). 2020 Mar 19;12(3):722. doi: 10.3390/cancers12030722 (PMC7140033; doi:10.3390/cancers12030722)
Supplement: Supplementary file 1 [file cancers-12-00722-s001.zip › suppl table and figures proof/Cancers All Suppl figures proof.pptx]

## Slide 1
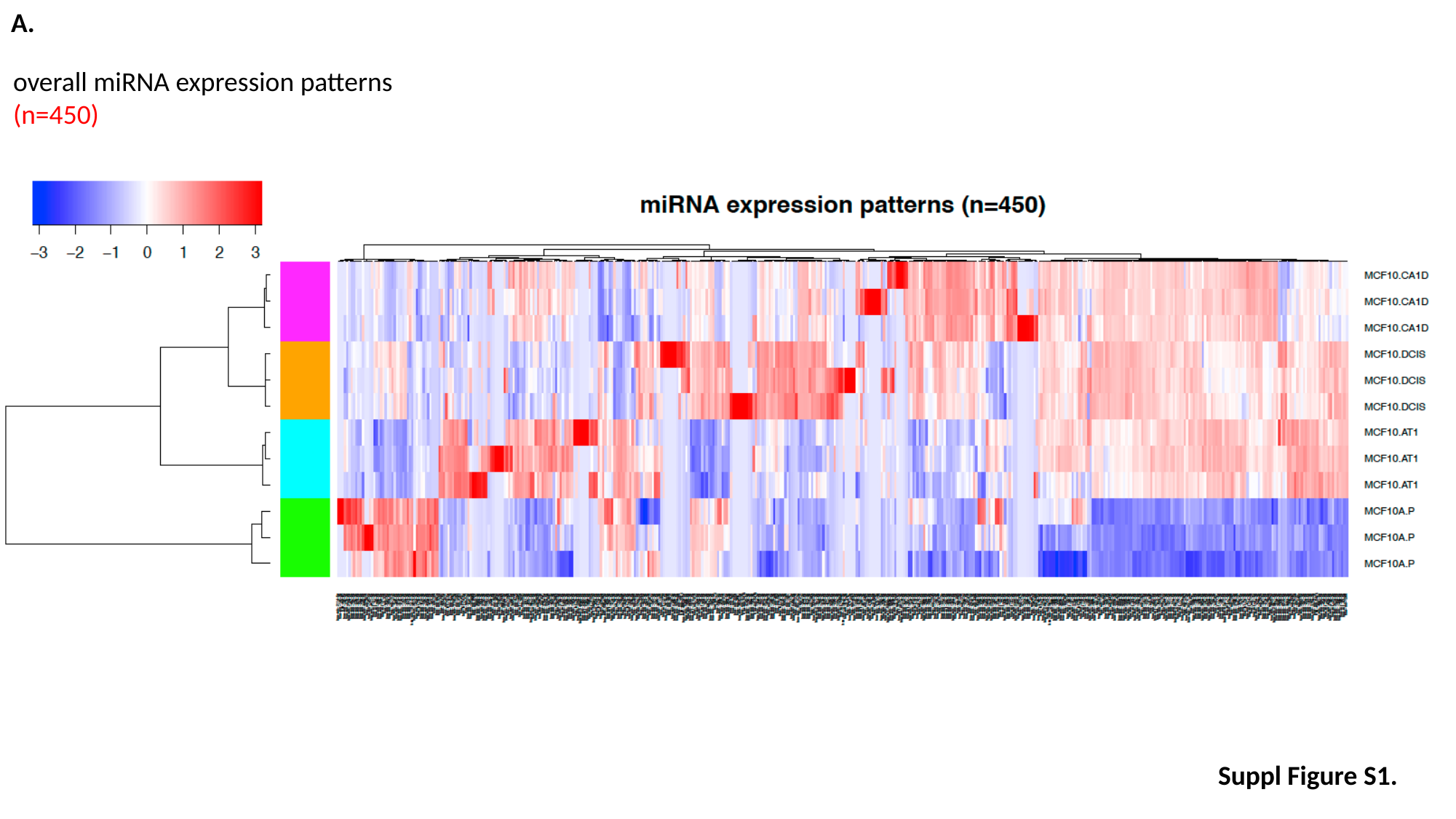

A.
overall miRNA expression patterns
(n=450)
Suppl Figure S1.

## Slide 2
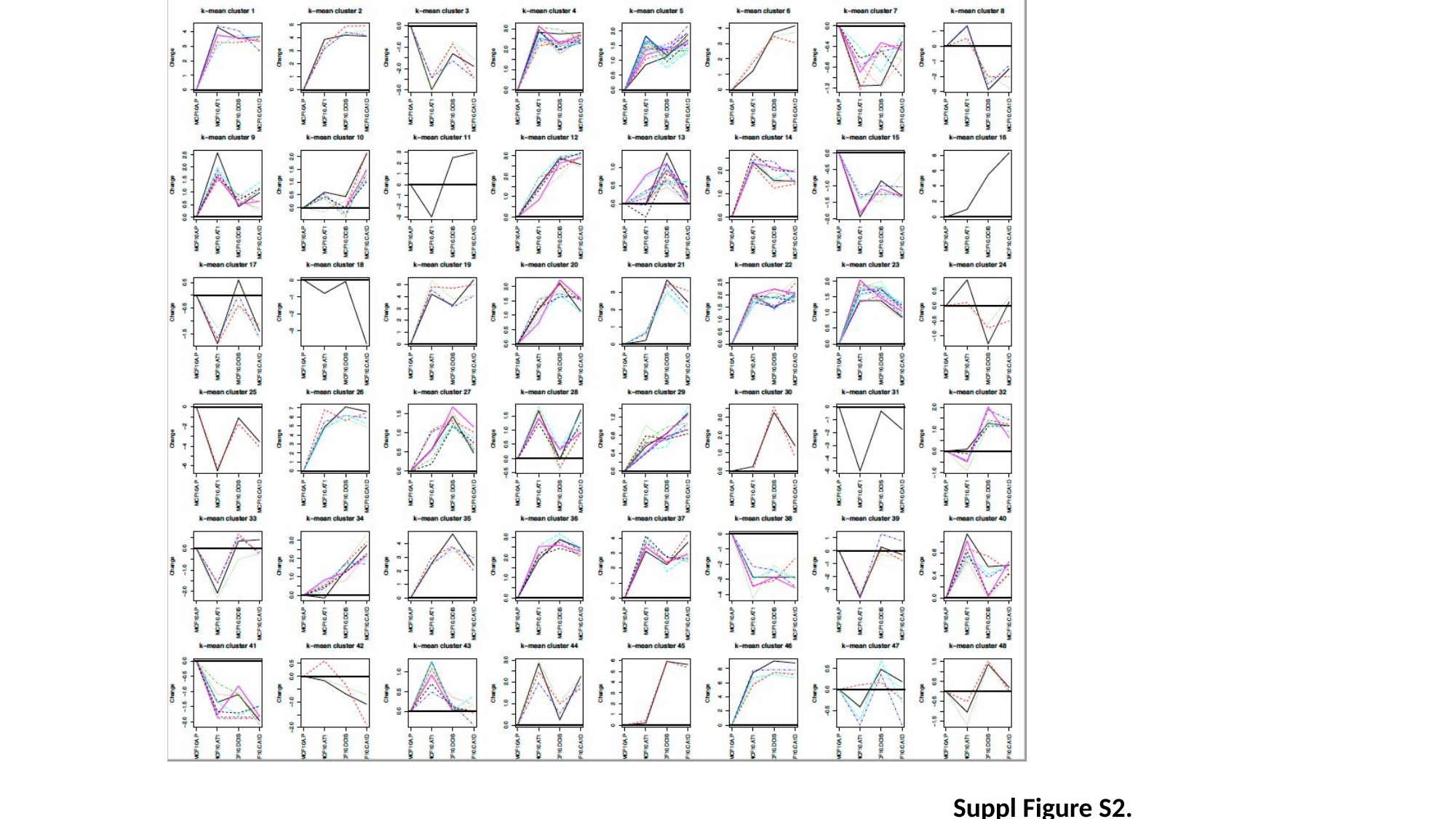

Suppl Figure S2.

## Slide 3
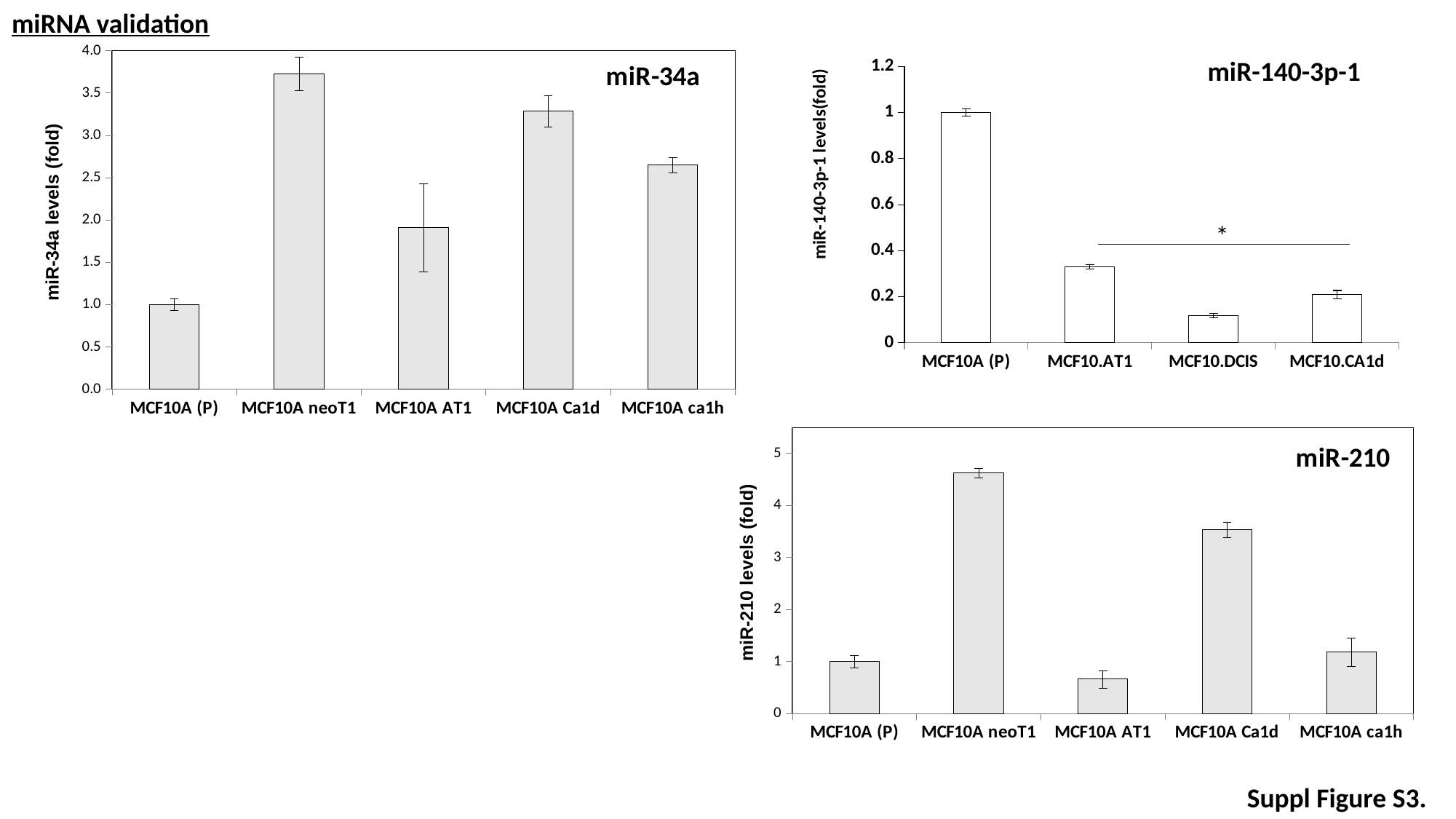

miRNA validation
### Chart: miR-34a
| Category | |
|---|---|
| MCF10A (P) | 1.0 |
| MCF10A neoT1 | 3.727 |
| MCF10A AT1 | 1.91 |
| MCF10A Ca1d | 3.284 |
| MCF10A ca1h | 2.647 |miR-140-3p-1
### Chart
| Category | Folds |
|---|---|
| MCF10A (P) | 1.0 |
| MCF10.AT1 | 0.331 |
| MCF10.DCIS | 0.118 |
| MCF10.CA1d | 0.209 |miR-140-3p-1 levels(fold)
 miR-34a levels (fold)
*
### Chart: miR-210
| Category | |
|---|---|
| MCF10A (P) | 1.0 |
| MCF10A neoT1 | 4.619999999999996 |
| MCF10A AT1 | 0.66 |
| MCF10A Ca1d | 3.533 |
| MCF10A ca1h | 1.177 | miR-210 levels (fold)
Suppl Figure S3.

## Slide 4
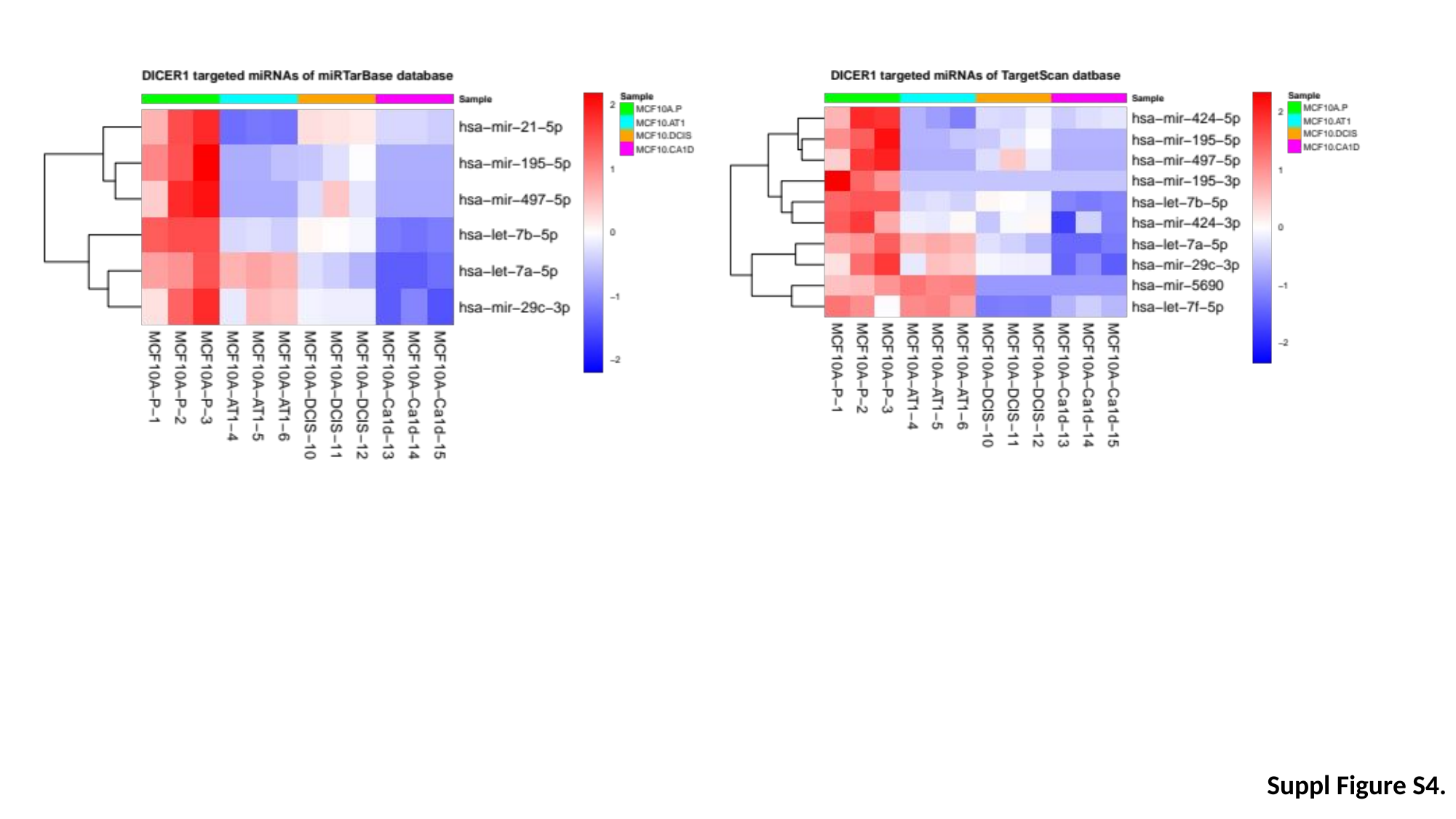

Suppl Figure S4.

## Slide 5
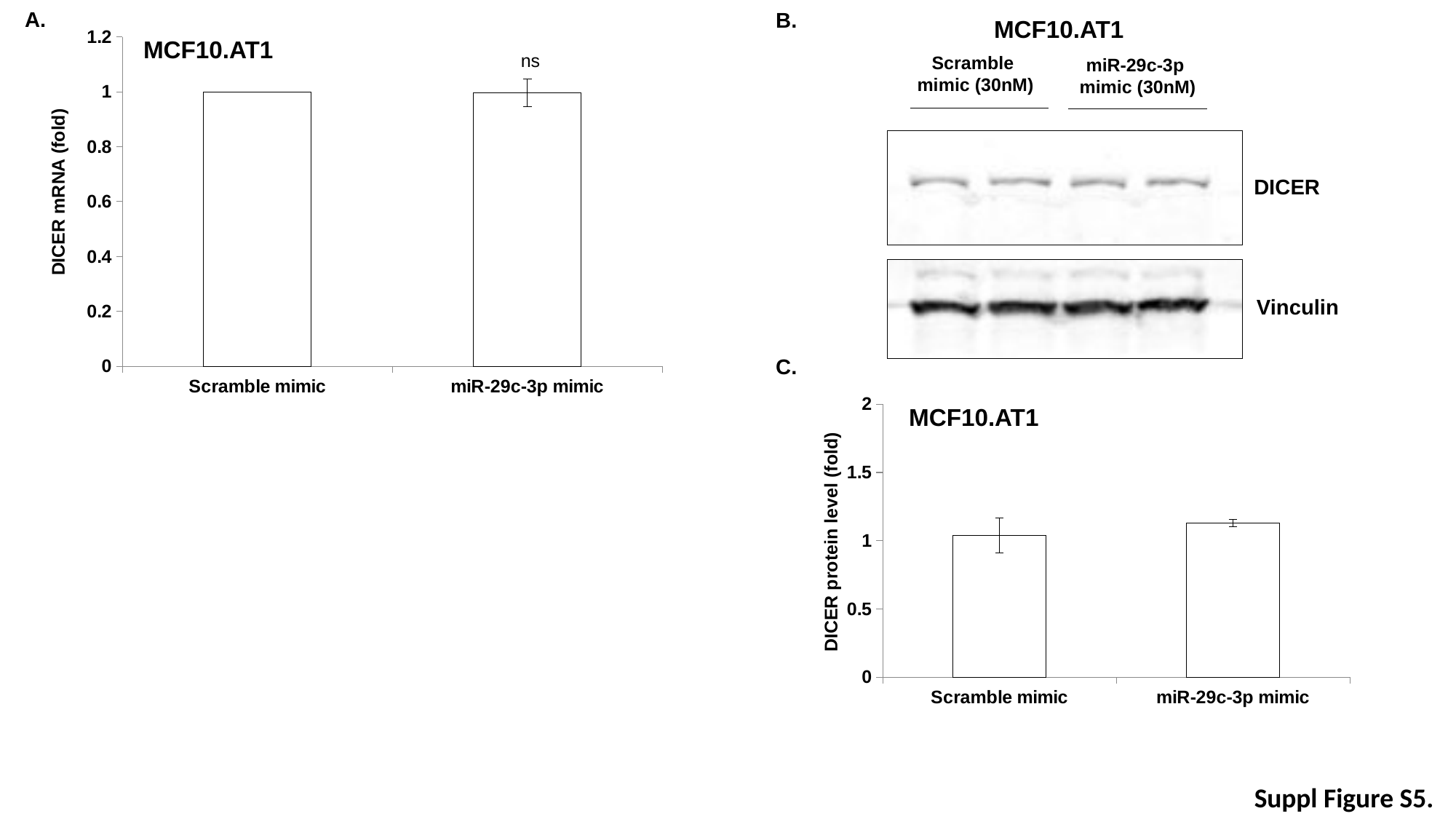

A.
B.
MCF10.AT1
### Chart
| Category | DICER mRNA |
|---|---|
| Scramble mimic | 1.0 |
| miR-29c-3p mimic | 0.996 |MCF10.AT1
ns
Scramble
mimic (30nM)
miR-29c-3p
mimic (30nM)
DICER
DICER mRNA (fold)
Vinculin
C.
### Chart
| Category | Mean |
|---|---|
| Scramble mimic | 1.04 |
| miR-29c-3p mimic | 1.13 |MCF10.AT1
DICER protein level (fold)
Suppl Figure S5.

## Slide 6
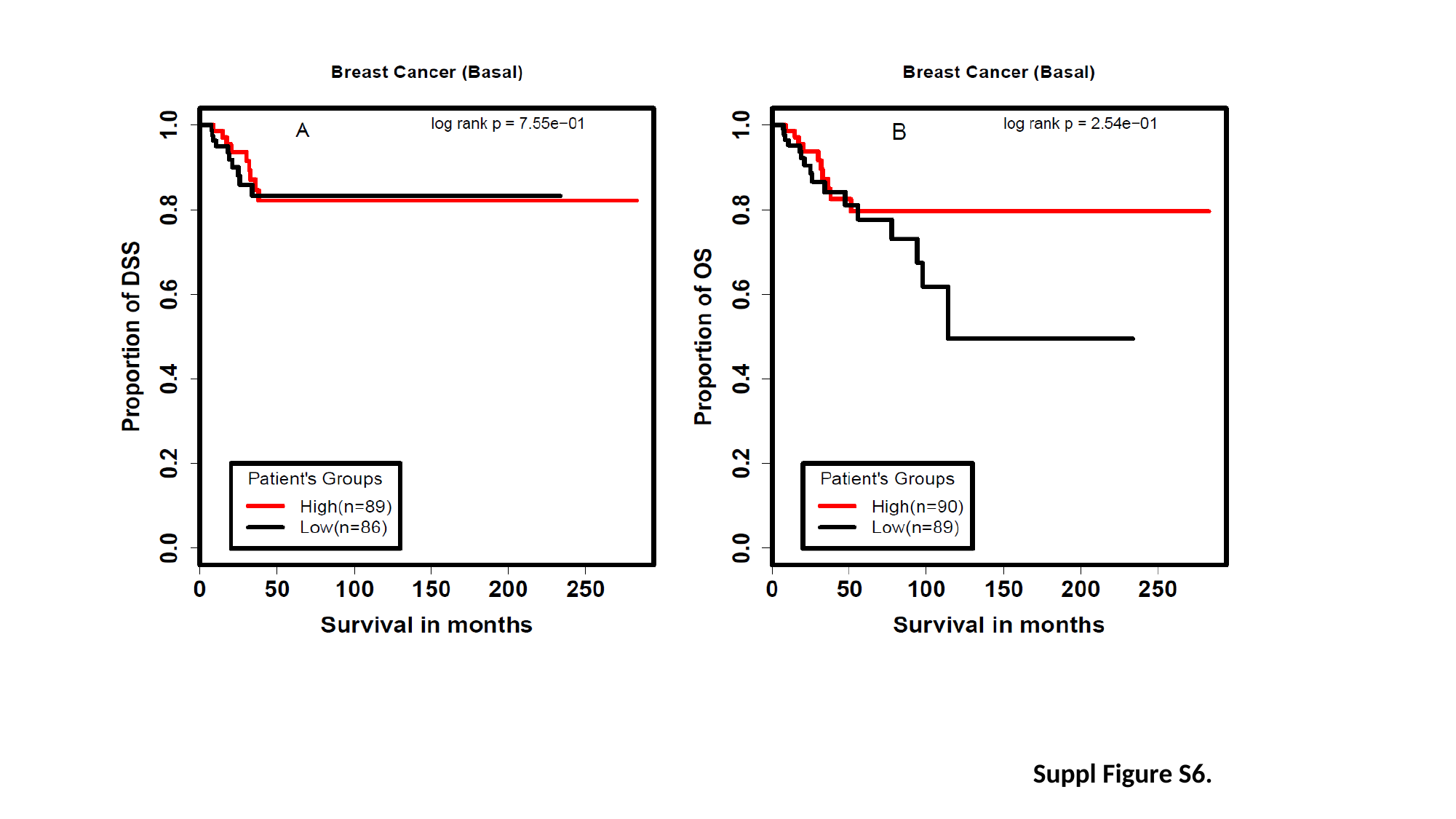

Suppl Figure S6.

## Slide 7
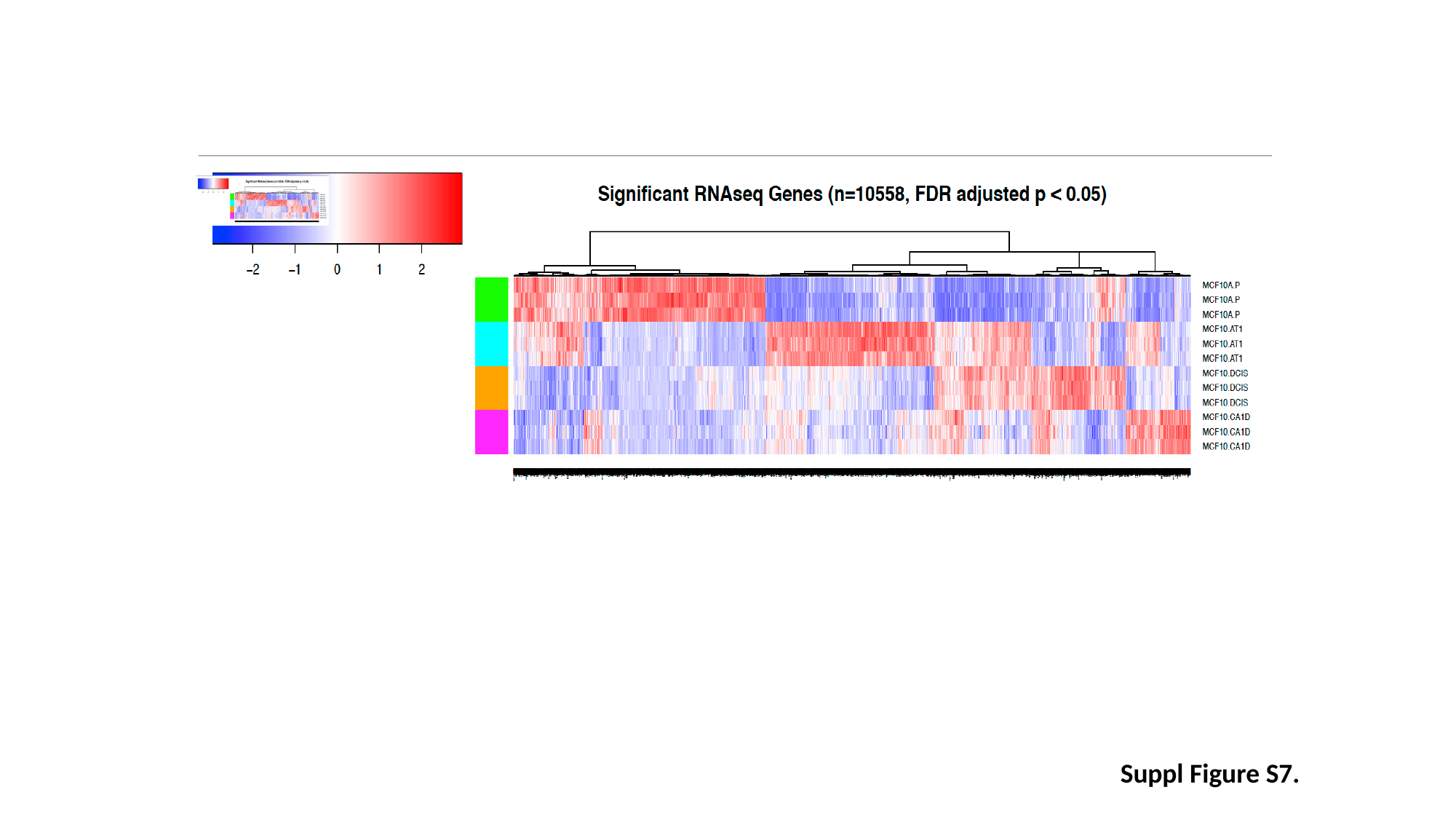

Suppl Figure S7.

## Slide 8
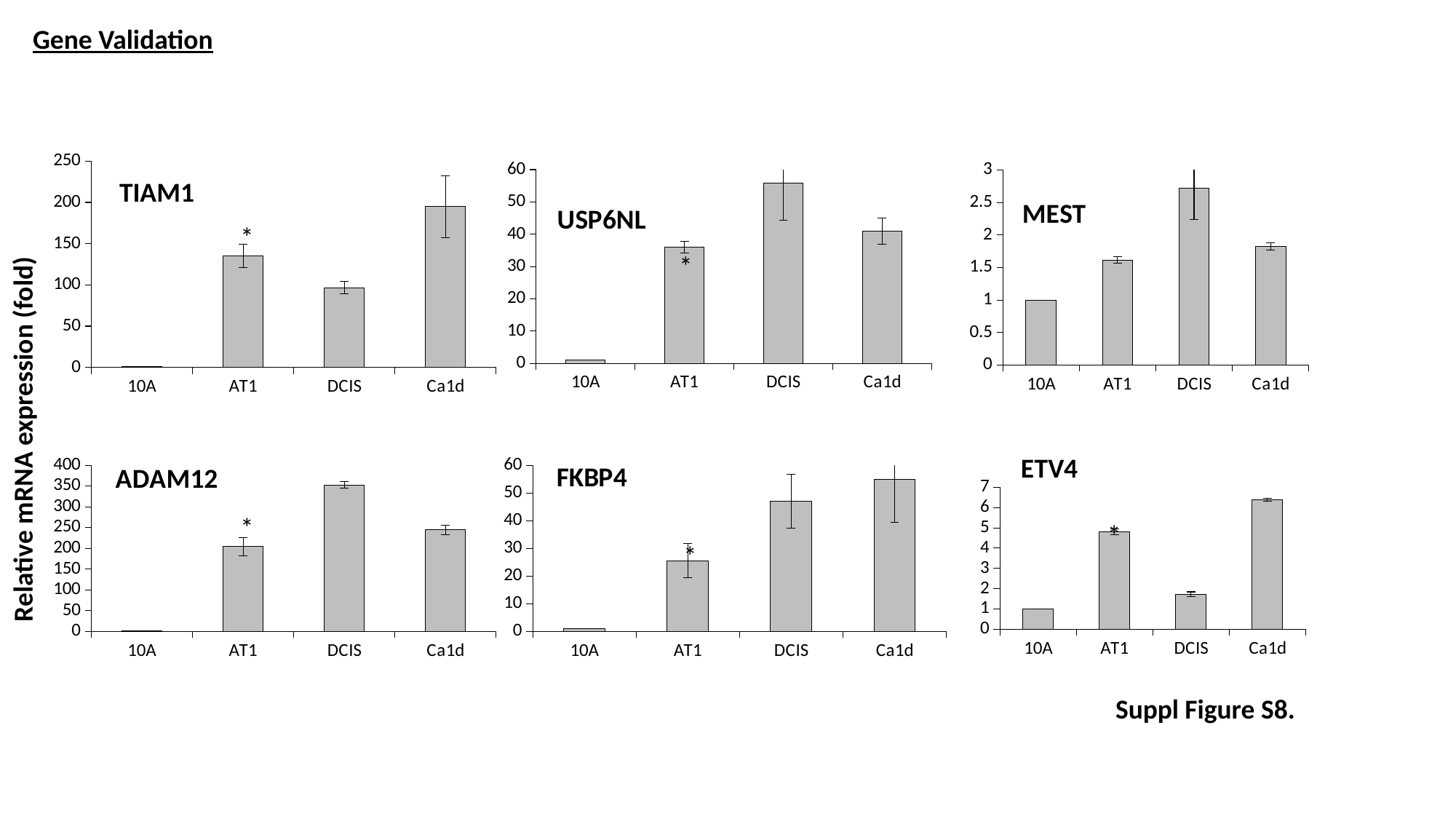

Gene Validation
### Chart:
| Category | USP6NL |
|---|---|
| 10A | 1.0 |
| AT1 | 36.00650411007823 |
| DCIS | 55.9106968355642 |
| Ca1d | 40.98716782410511 |
### Chart
| Category | TIAN1 |
|---|---|
| 10A | 1.0 |
| AT1 | 135.0885866212121 |
| DCIS | 96.51730888577576 |
| Ca1d | 194.9726404793207 |
### Chart:
| Category | MEST |
|---|---|
| 10A | 1.0 |
| AT1 | 1.616135588180217 |
| DCIS | 2.715841428424464 |
| Ca1d | 1.825483893544225 |TIAM1
*
*
### Chart:
| Category | ADAM12 |
|---|---|
| 10A | 1.0 |
| AT1 | 204.1069560581452 |
| DCIS | 352.599043759897 |
| Ca1d | 244.3522213752357 |
### Chart:
| Category | FKBP4 |
|---|---|
| 10A | 1.0 |
| AT1 | 25.54578401509115 |
| DCIS | 47.04034662828773 |
| Ca1d | 54.9478720398255 |
### Chart:
| Category | ETV4 |
|---|---|
| 10A | 1.0 |
| AT1 | 4.805918221040033 |
| DCIS | 1.73374451833406 |
| Ca1d | 6.39210020102614 |Relative mRNA expression (fold)
*
*
*
Suppl Figure S8.

## Slide 9
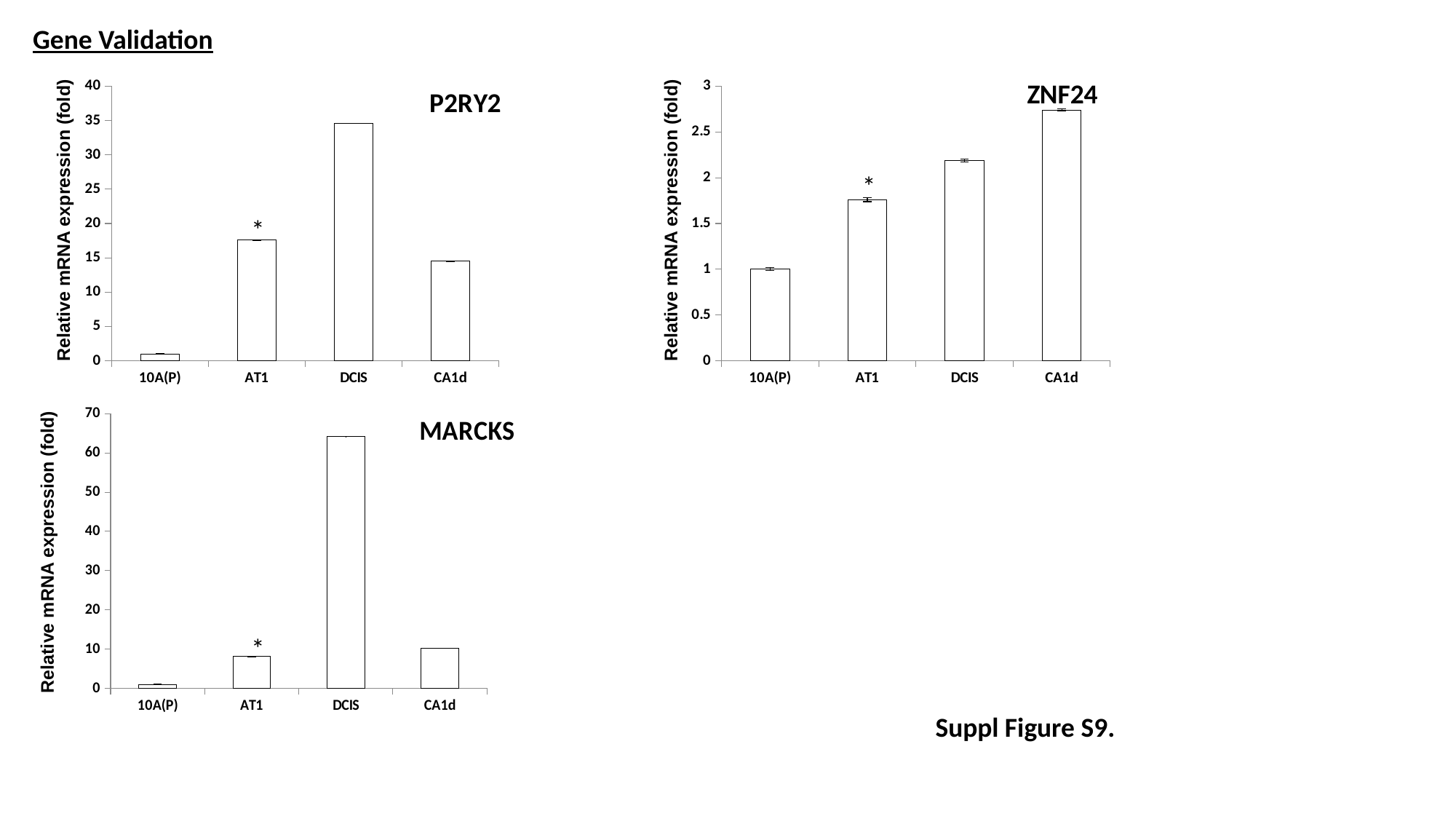

Gene Validation
### Chart: P2RY2
| Category | P2RY2 |
|---|---|
| 10A(P) | 1.0 |
| AT1 | 17.567 |
| DCIS | 34.59 |
| CA1d | 14.497 |
### Chart: ZNF24
| Category | ZNF24 |
|---|---|
| 10A(P) | 1.0 |
| AT1 | 1.76 |
| DCIS | 2.19 |
| CA1d | 2.739 |*
Relative mRNA expression (fold)
Relative mRNA expression (fold)
*
### Chart: MARCKS
| Category | MARKCS |
|---|---|
| 10A(P) | 1.0 |
| AT1 | 8.08 |
| DCIS | 64.2 |
| CA1d | 10.2 |Relative mRNA expression (fold)
*
Suppl Figure S9.

## Slide 10
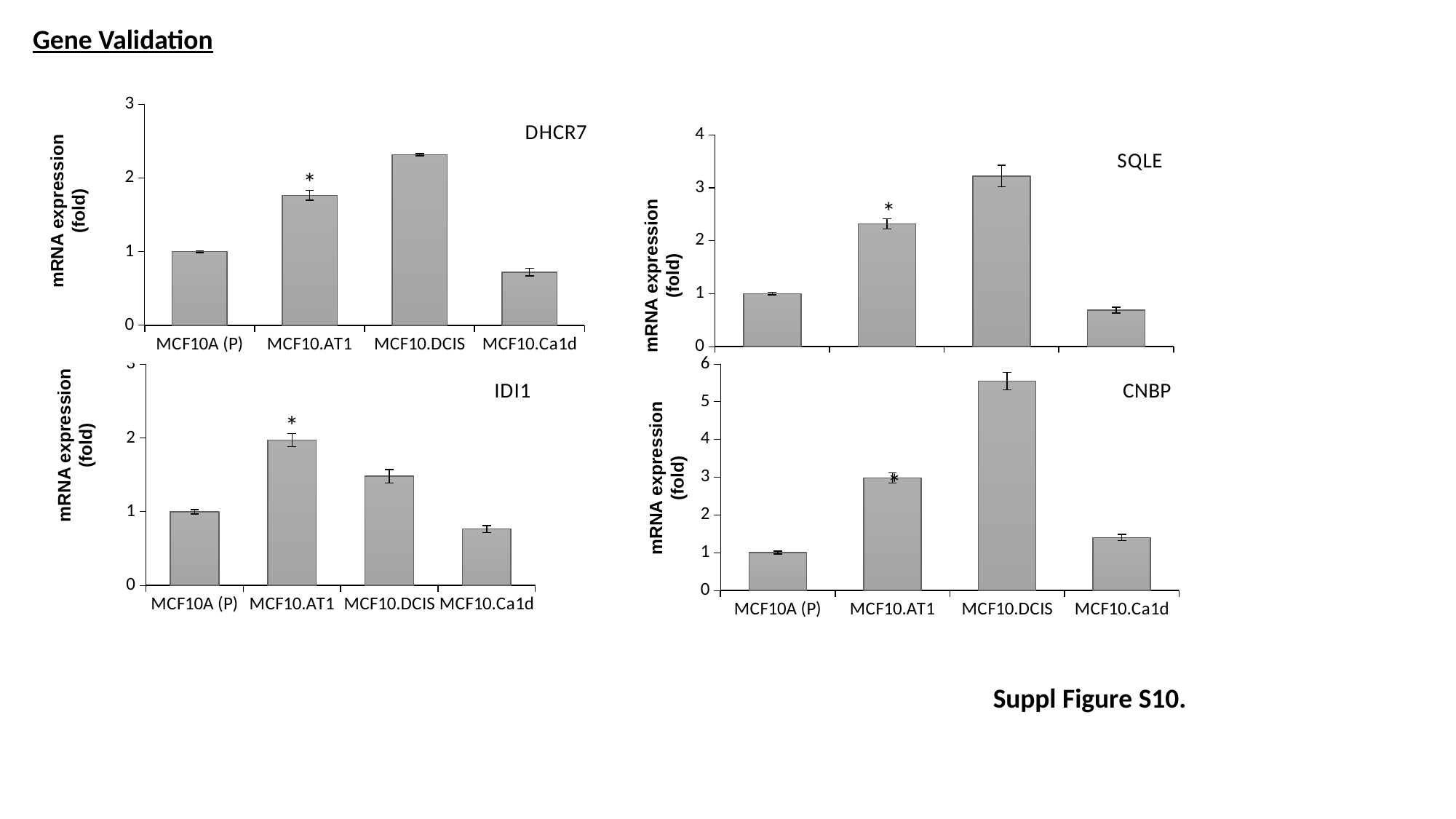

Gene Validation
### Chart: DHCR7
| Category | DHCR7 |
|---|---|
| MCF10A (P) | 1.0 |
| MCF10.AT1 | 1.76628329843875 |
| MCF10.DCIS | 2.317946804664626 |
| MCF10.Ca1d | 0.721710893437566 |
### Chart: SQLE
| Category | SQLE |
|---|---|
| MCF10A (P) | 1.0 |
| MCF10.AT1 | 2.319470389958288 |
| MCF10.DCIS | 3.223569908939438 |
| MCF10.Ca1d | 0.688289684239324 |*
mRNA expression (fold)
*
mRNA expression (fold)
### Chart: CNBP
| Category | CNBP |
|---|---|
| MCF10A (P) | 1.0 |
| MCF10.AT1 | 2.982014311140954 |
| MCF10.DCIS | 5.550890353098405 |
| MCF10.Ca1d | 1.402704988511548 |
### Chart: IDI1
| Category | IDI1 |
|---|---|
| MCF10A (P) | 1.0 |
| MCF10.AT1 | 1.973710065694109 |
| MCF10.DCIS | 1.482556897562238 |
| MCF10.Ca1d | 0.766590303047487 |*
mRNA expression (fold)
mRNA expression (fold)
*
Suppl Figure S10.

## Slide 11
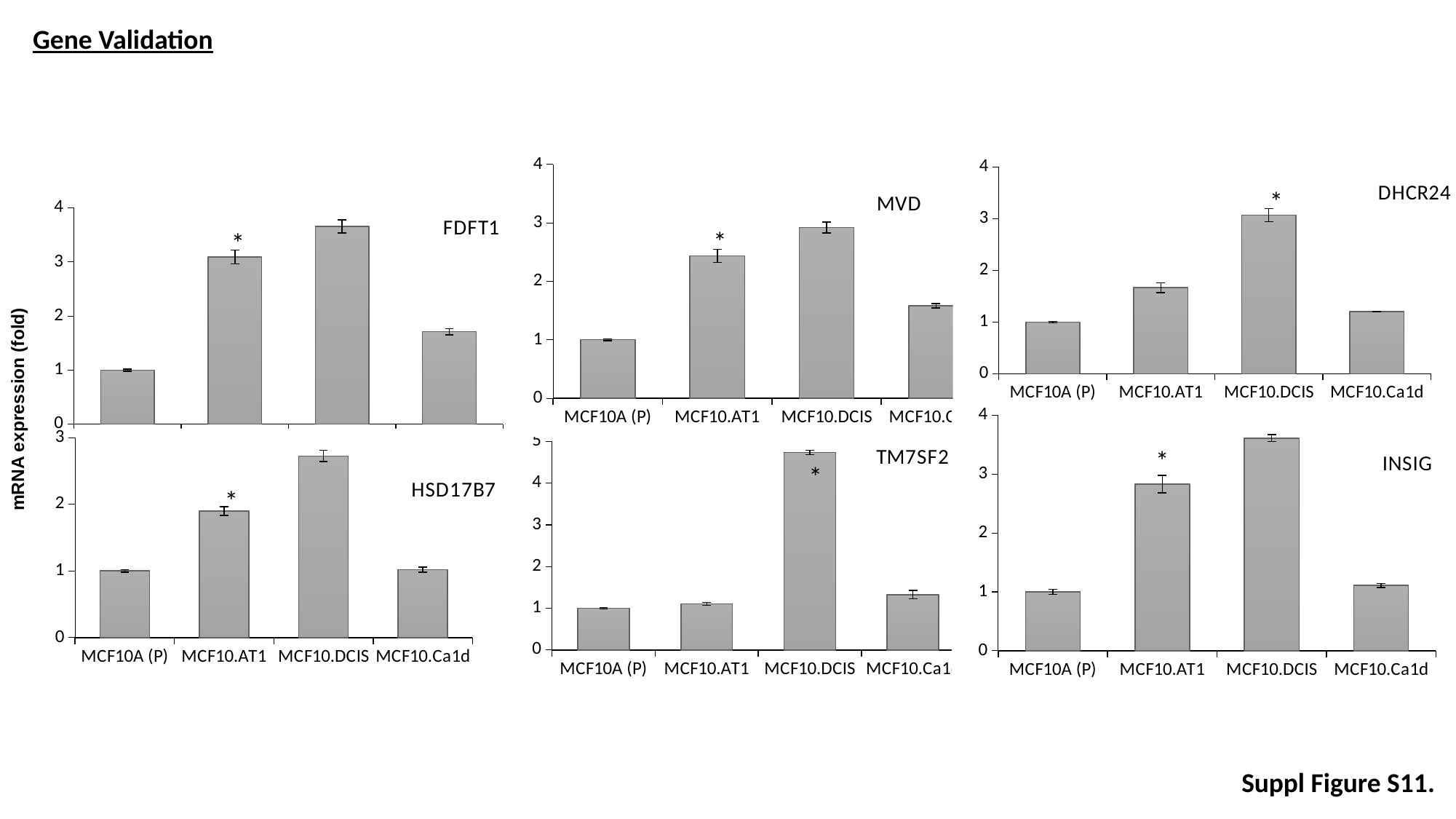

Gene Validation
### Chart: MVD
| Category | MVD |
|---|---|
| MCF10A (P) | 1.0 |
| MCF10.AT1 | 2.433525047195362 |
| MCF10.DCIS | 2.920828624514026 |
| MCF10.Ca1d | 1.580168379984481 |
### Chart: DHCR24
| Category | DHCR24 |
|---|---|
| MCF10A (P) | 1.0 |
| MCF10.AT1 | 1.663546257244425 |
| MCF10.DCIS | 3.070759161660341 |
| MCF10.Ca1d | 1.20006856747423 |*
### Chart: FDFT1
| Category | FDFT1 |
|---|---|
| MCF10A (P) | 1.0 |
| MCF10.AT1 | 3.092035208051062 |
| MCF10.DCIS | 3.658797080855691 |
| MCF10.Ca1d | 1.709808604029513 |*
*
mRNA expression (fold)
### Chart: INSIG
| Category | INSIG |
|---|---|
| MCF10A (P) | 1.0 |
| MCF10.AT1 | 2.832753276478276 |
| MCF10.DCIS | 3.615520241558062 |
| MCF10.Ca1d | 1.10999616946327 |
### Chart: HSD17B7
| Category | HSD17B7 |
|---|---|
| MCF10A (P) | 1.0 |
| MCF10.AT1 | 1.895277381040281 |
| MCF10.DCIS | 2.724108195581334 |
| MCF10.Ca1d | 1.020688445905473 |
### Chart: TM7SF2
| Category | TM7SF2 |
|---|---|
| MCF10A (P) | 1.0 |
| MCF10.AT1 | 1.103220404135447 |
| MCF10.DCIS | 4.73918164578854 |
| MCF10.Ca1d | 1.326326254033489 |*
*
*
Suppl Figure S11.

## Slide 12
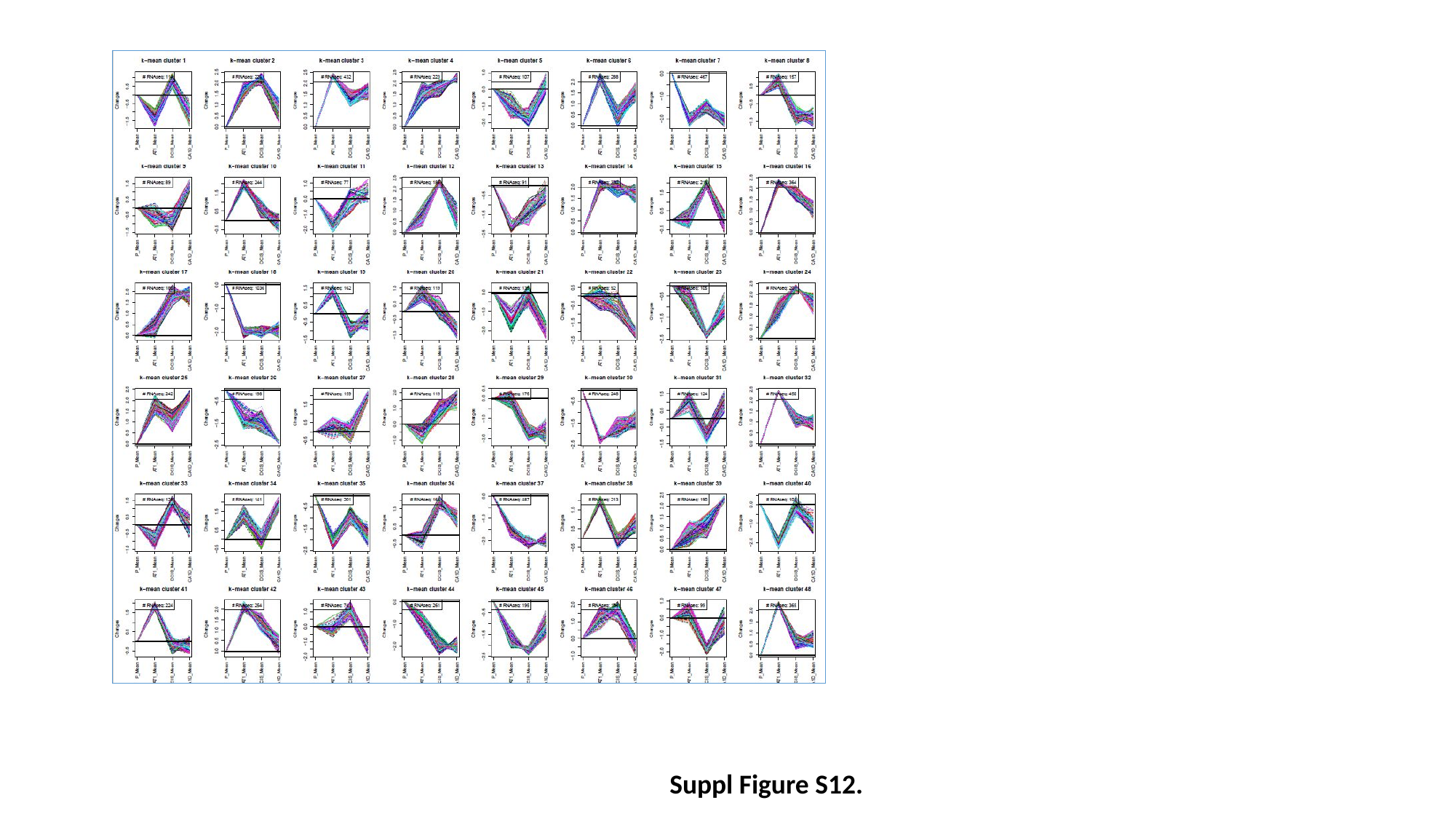

Suppl Figure S12.

## Slide 13
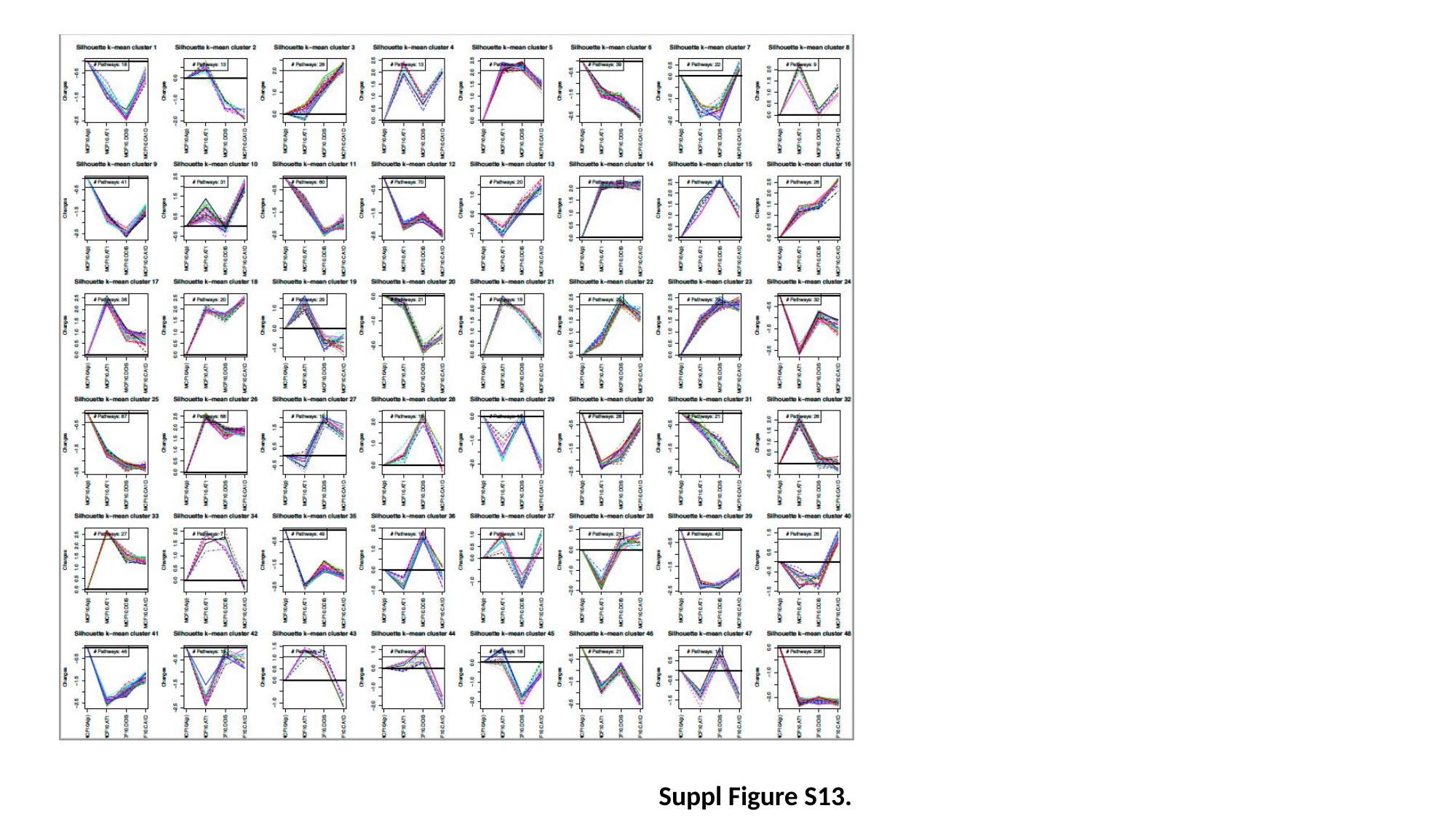

Suppl Figure S13.

## Slide 14
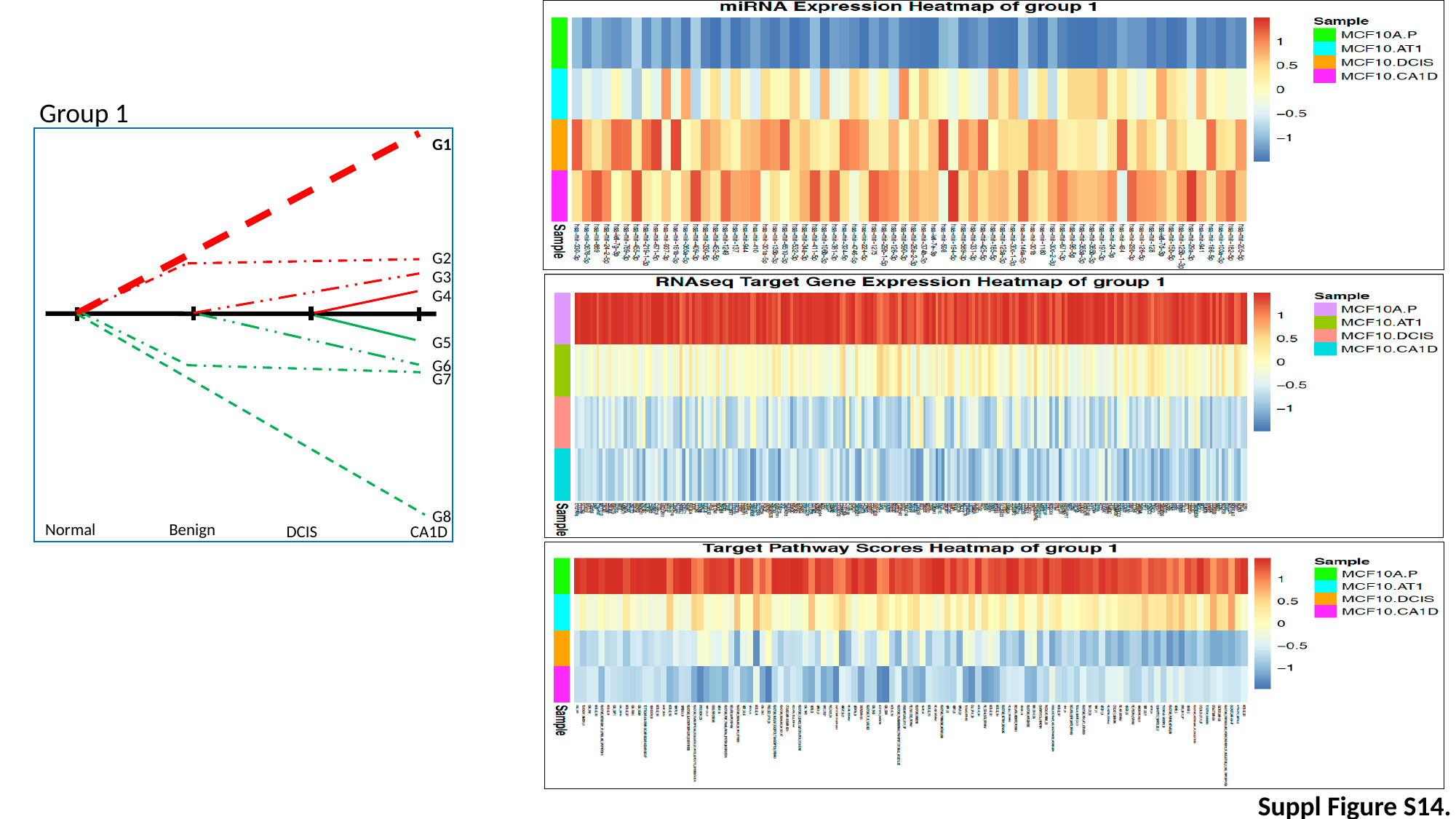

Group 1
G1
G2
G3
G4
G5
G6
G7
G8
Normal
Benign
DCIS
CA1D
Suppl Figure S14.

## Slide 15
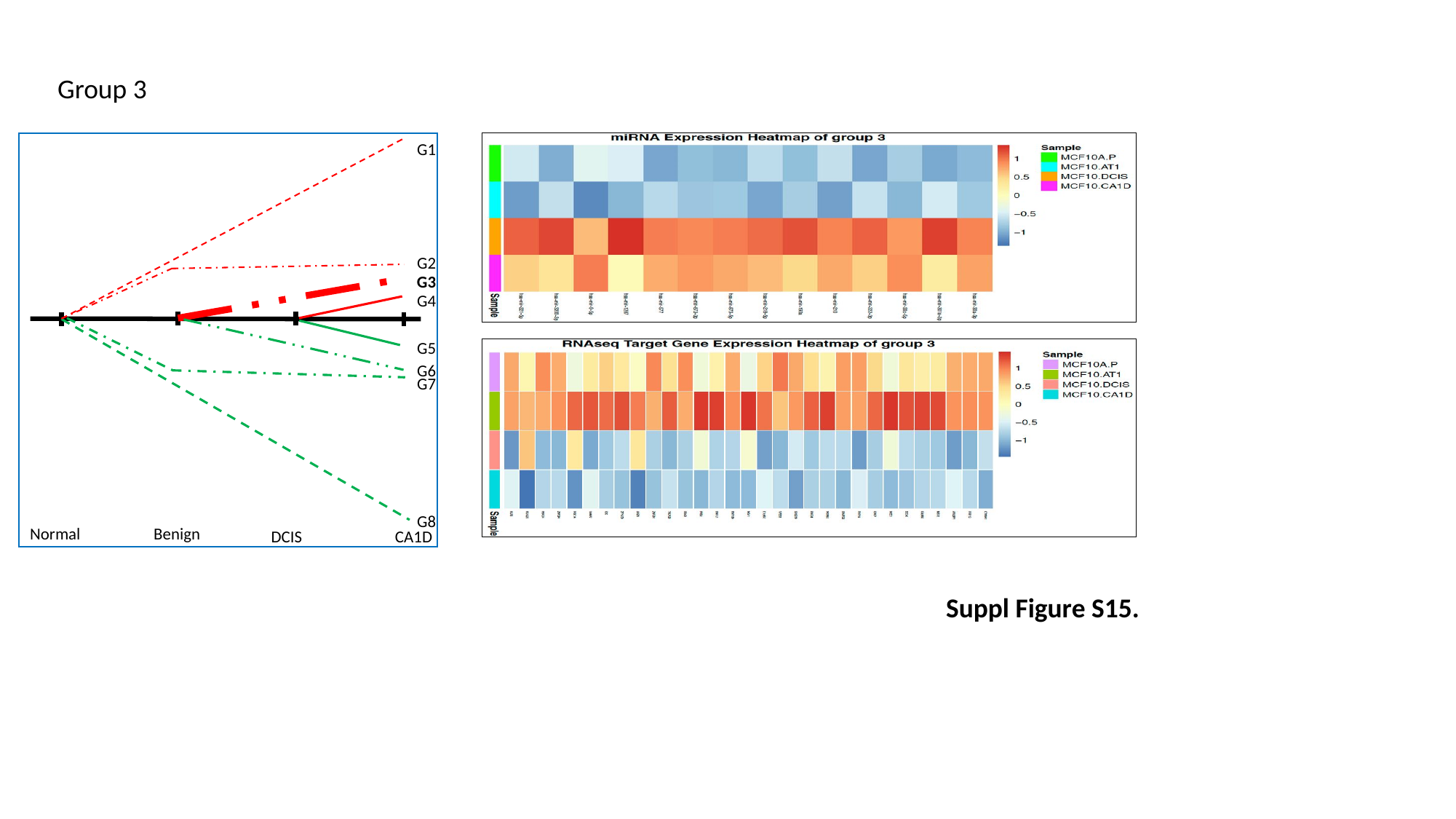

Group 3
G1
G2
G3
G4
G5
G6
G7
G8
Normal
Benign
DCIS
CA1D
Suppl Figure S15.

## Slide 16
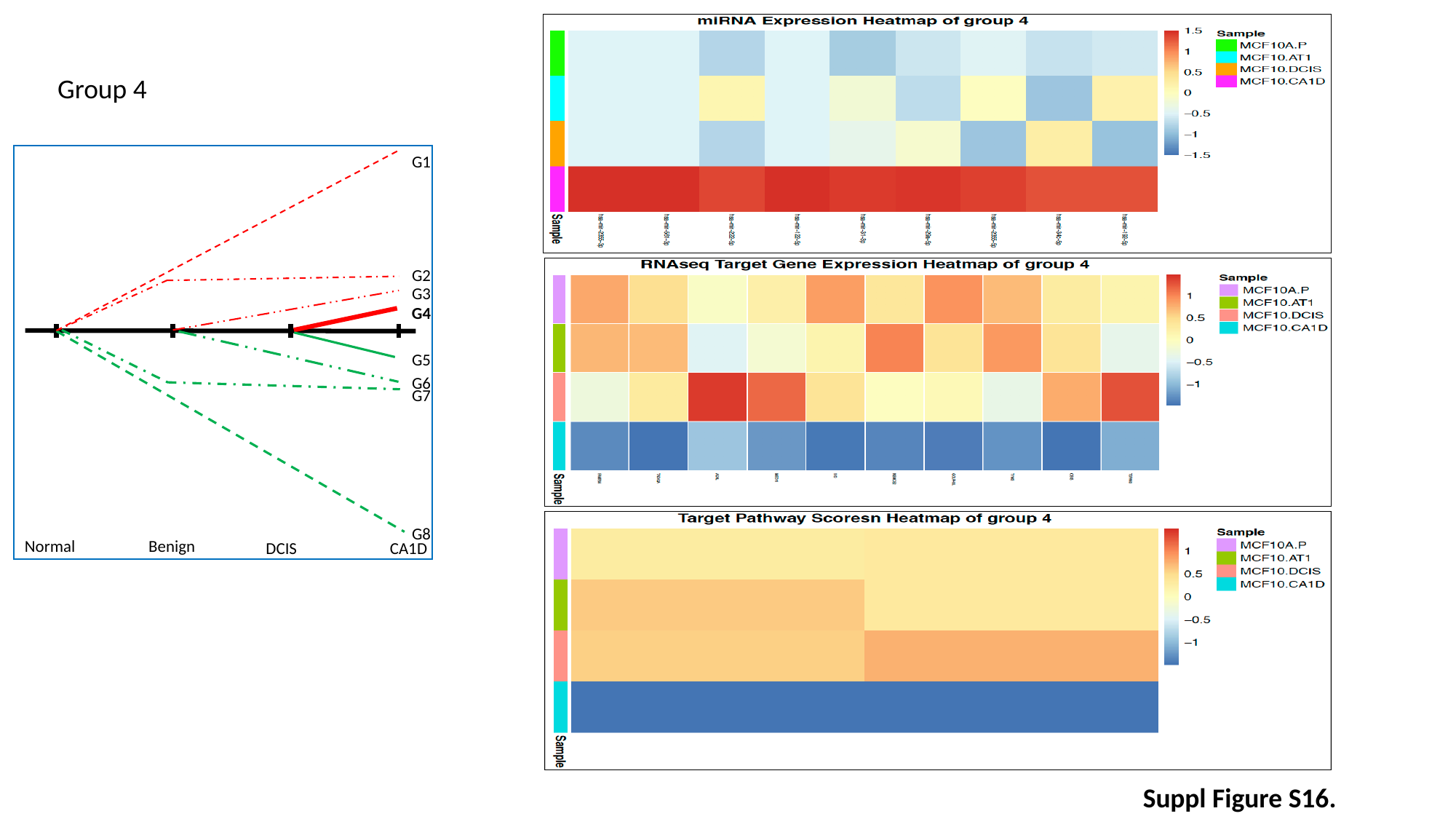

Group 4
G1
G2
G3
G4
G5
G6
G7
G8
Normal
Benign
DCIS
CA1D
Suppl Figure S16.

## Slide 17
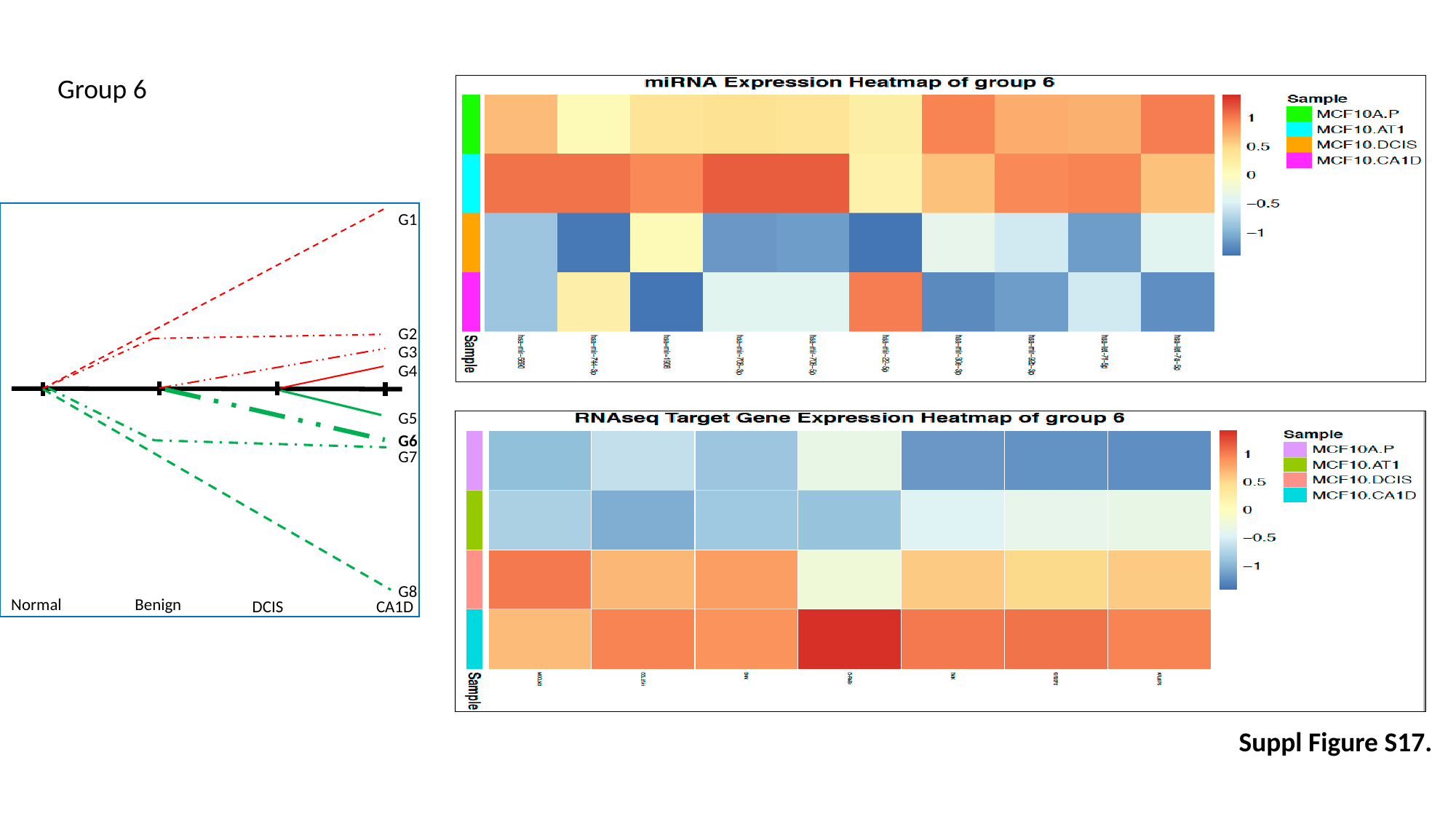

Group 6
G1
G2
G3
G4
G5
G6
G7
G8
Normal
Benign
DCIS
CA1D
Suppl Figure S17.

## Slide 18
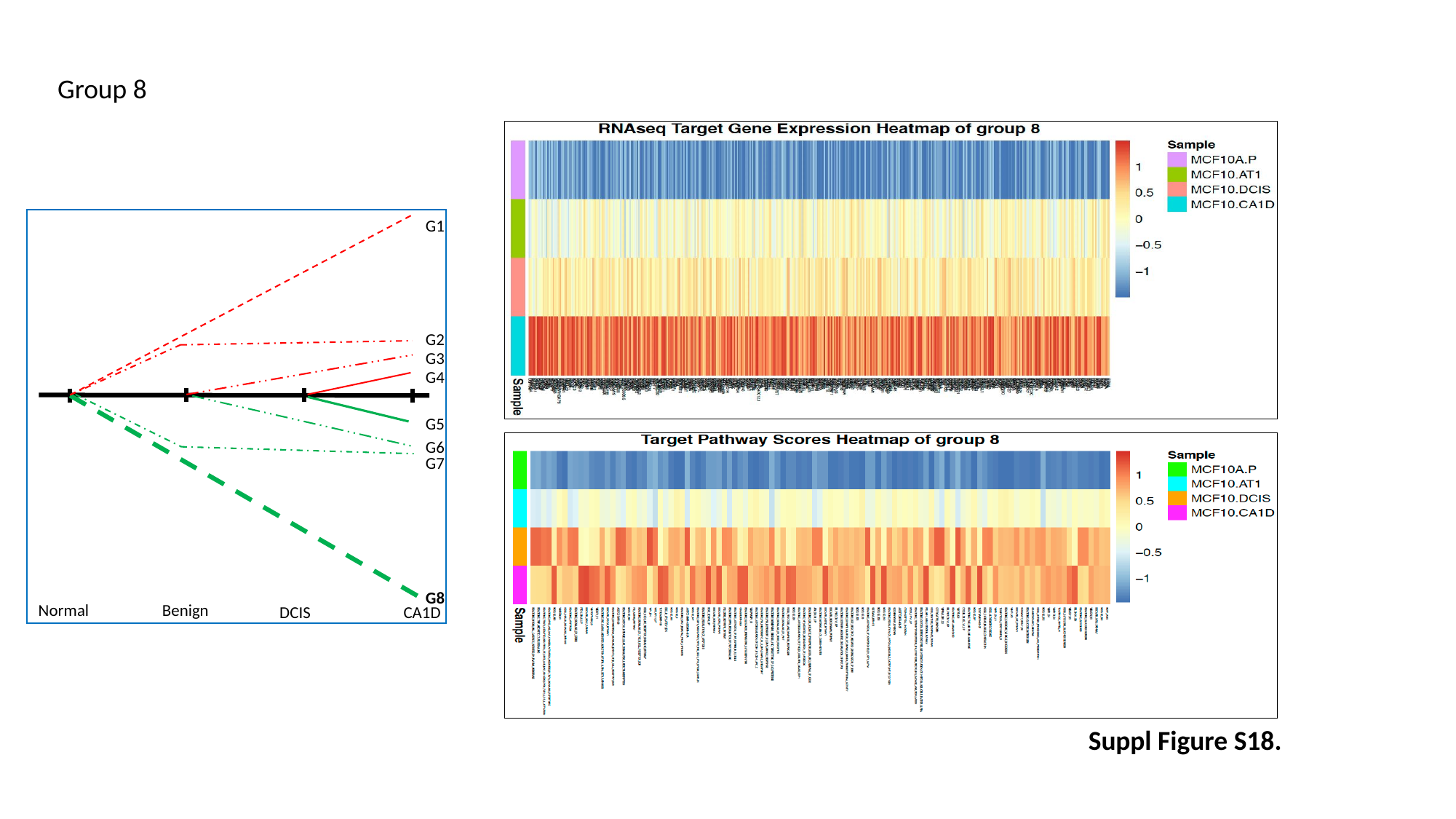

Group 8
G1
G2
G3
G4
G5
G6
G7
G8
Normal
Benign
DCIS
CA1D
Suppl Figure S18.
