# Supplementary material for: Integrative Analyses of Multilevel Omics Reveal Preneoplastic Breast to Possess a Molecular Landscape That is Globally Shared with Invasive Basal-Like Breast Cancer (Running Title: Molecular Landscape of Basal-Like Breast Cancer Progression)
Source: Cancers (Basel). 2020 Mar 19;12(3):722. doi: 10.3390/cancers12030722 (PMC7140033; doi:10.3390/cancers12030722)
Supplement: Supplementary file 1 [file cancers-12-00722-s001.zip › suppl table and figures proof/Cancers-Suppl figures-legends-Proof.docx]

**Supplementary Figures**

**Supplementary Figure S1: Heat map showing the expression pattern of all miRNAs (n=450) that changed across the spectrum of breast cancer progression** comprising normal (MCF10A.P), preneoplastic (MCF10.AT1), ductal carcinoma in situ (MCF10.DCIS), and invasive cancer (MCF10.CA1D) cells. The color key shows blue as lowest expression and red as highest expression.

**Supplementary Figure S2:** **Line diagrams showing the K-means clustering to group miRNAs in one of 8 predefined patterns (Fig. 1C) to reliably choose miRNAs of interest.** Embedded in each panel is the number of miRNAs that fell within the pattern shown.

**Supplementary Figure S3:** **qPCR validation of significantly changing miRNAs in the MCF10A-based breast cancer progression panel.** Relative mean endogenous expression of indicated miRNAs assayed by TaqMan-based qPCR across the breast cancer progression panel. miRNAs of interest were first normalized to a small nucleolar RNA, RNU44. The Ct values were converted to fold change using the delta Ct method and setting the lowest gene expression (MCF10A.P cells, in this case) as 1.

**Supplementary Figure S4:** Heat maps showing the expression pattern of mRNAs that change significantly across the MCF10A model of breast cancer development and are predicted regulators of DICER by miRTarBase and TargetScan algorithms. The color key shows blue as lowest expression and red as highest expression.

**Supplementary Figure S5:** **miRNA-29c-3p does not regulate DICER. (A)** Relative mean expression of endogenous *DICER1* mRNA assayed by qPCR in MCF10.AT1 cells transfected with either scramble mimic or miR-29c-3p mimics. *DICER1* mRNA levels were first normalized to a housekeeping gene, RPL19. The Ct values in miR-29c-3p mimic-treated cells were converted to fold change using the delta Ct method and setting the gene expression in scramble-treated MCF10.AT1 cells as 1. **(B)** Western blot analysis and **(C)** its quantification showing no change in the expression of endogenous *DICER1* protein caused by miR-29c-3p mimics compared to scramble control mimics in preneoplastic MCF10.AT1 cells. DICER protein levels were normalized to a loading control, vinculin. The graphs represent mean values + SEM. **(**P values were calculated using students’ unpaired t test. * indicates p<0.05.


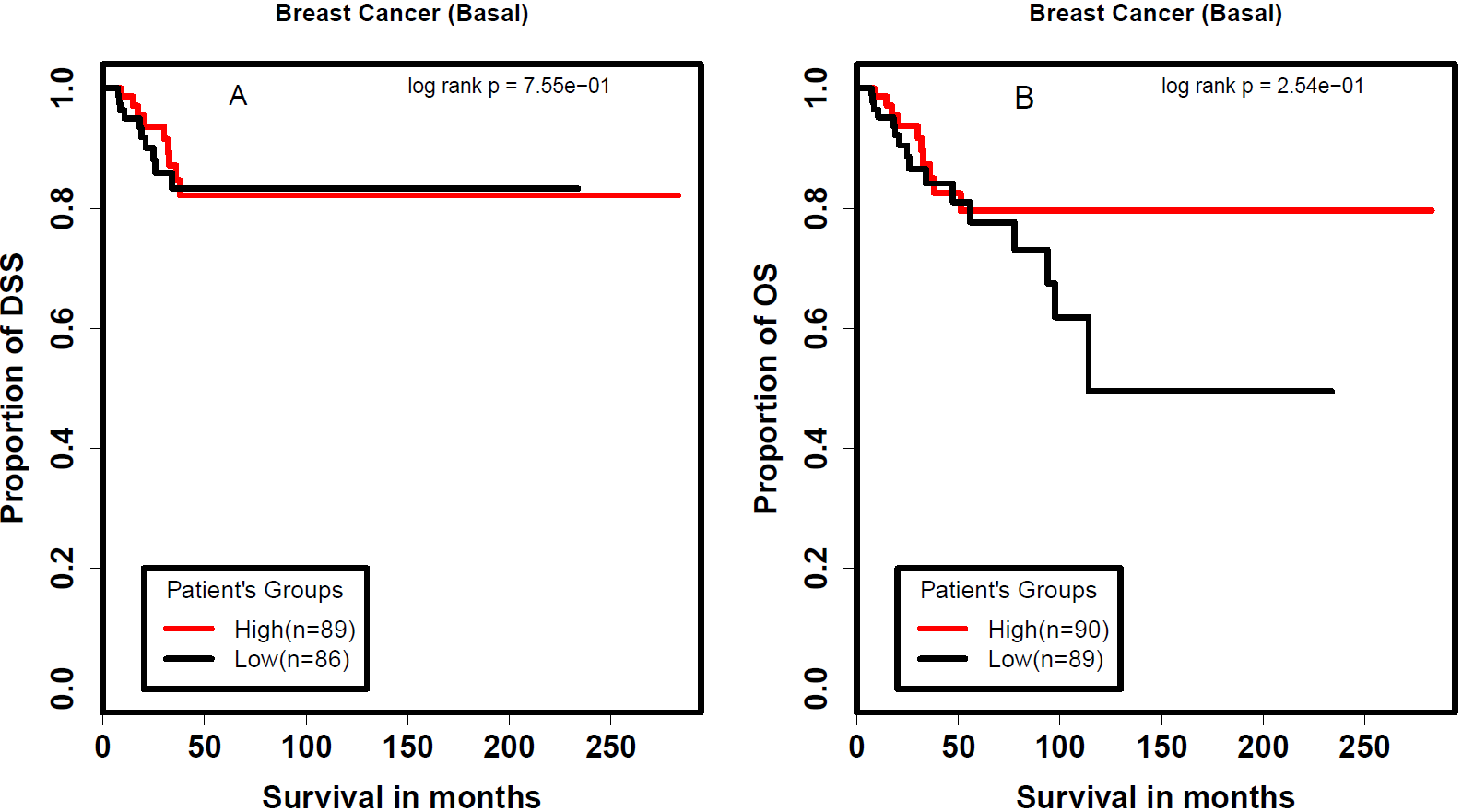


**Supplementary Figure S6:** **let-7b-5p and prognosis in basal like breast cancer subtype patients.** Kaplan-Meier curves showing **(A)** disease-specific survival (in months) and **(B)** overall survival in breast cancer patients expressing high vs low levels of let-7b-5p.

**Supplementary Figure S7:** Heat map showing the expression pattern of mRNAs that change significantly across the MCF10A model of breast cancer development. The color key shows blue as lowest expression and red as highest expression.

**Supplementary Figures S8-S11**

**Supplementary Figure S8-11:** **qPCR validation of significantly changing genes in the MCF10A-based breast cancer progression panel.** Relative mean endogenous expression of indicated mRNAs assayed by qPCR across the breast cancer progression panel. mRNAs of interest were first normalized to a housekeeping gene, RPL19. The Ct values were converted to fold change using the delta Ct method and setting the lowest gene expression (MCF10A.P cells, in this case) as 1.

**Supplementary Figure S12:** Line diagrams showing the K-means clustering to group mRNAs in one of the 8 predefined patterns (Fig. 1C) to reliably choose mRNAs of interest. Embedded in each panel is the number of mRNAs that fell within the pattern shown.

**Supplementary Figure S13:** Line diagrams showing the K-means clustering to group pathways in one of the 8 predefined patterns (Fig. 1C). Embedded in each panel is the number of pathways that fell within the pattern shown.

**Supplementary Figure S14:** **miRNAs that increased continuously and their target genes and** t**arget pathways** **that decreased** **continuously** **during breast cancer progression (Group 1).** Heat maps showing miRNAs and their target genes and pathways that mapped to Group 1 represent early-continued increase in miRNA and decrease in target genes and pathways across the normal-like to invasive transition of breast cancer progression. The color key shows blue as lowest expression and red as highest expression.

**Supplementary Figure S15:** **miRNAs that increased and their target genes that decreased** **during preneoplastic to invasive transition in breast cancer progression (Group 3).** Heatmaps showing miRNAs, and their target genes that mapped to Group 3 represent a delayed -continued increase in miRNA and decrease in target genes across the preneoplastic to invasive transition of breast cancer progression. The color key shows the blue color as lowest expression and red color as highest expression.

**Supplementary Figure S16:** **miRNA that increased, and their target genes &** t**arget pathways** **that decreased** **during DCIS to invasive transition in breast cancer progression (Group 4).** Heatmaps showing miRNAs, and their target genes that mapped to Group 4 represent a late increase in miRNA and decrease in target genes and pathways across the DCIS to invasive transition of breast cancer progression. The color key shows the blue color as lowest expression and red color as highest expression.

**Supplementary Figure S17:** **miRNA that decreased, and their target genes that increased** **during preneoplastic to invasive transition in breast cancer progression (Group 6).** Heatmaps showing miRNAs, and their target genes that mapped to Group 6 represent a delayed -continued decrease in miRNA and decrease in target genes across the preneoplastic to invasive transition of breast cancer progression. The color key shows the blue color as lowest expression and red color as highest expression.

**Supplementary Figure S18:** **miRNA that decreased, and their target genes that increased** **continuously in breast cancer progression (Group 8).** Heatmaps showing miRNAs, their target genes that mapped to Group 8 represent an early and continuous decrease in miRNA and increase in target genes across the normal- like to invasive transition of breast cancer progression. The color key shows the blue color as lowest expression and red color as highest expression.
